# MiR-27a-3p binds to TET1 mediated DNA demethylation of ADCY6 regulates breast cancer progression *via* epithelial-mesenchymal transition

**DOI:** 10.3389/fonc.2022.957511

**Published:** 2022-08-01

**Authors:** Hao Wu, Juanjuan Qiu, Zhenru Wu, Tao He, Chen Zhou, Qing Lv

**Affiliations:** ^1^ Department of Breast Surgery, West China Hospital, Sichuan University, Chengdu, China; ^2^ Laboratory of Pathology, West China Hospital, Sichuan University, Chengdu, China

**Keywords:** MiR-27a-3p, TET1, DNA methylation, ADCY6, breast cancer, epithelial-mesenchymal transition, tumor progression

## Abstract

**Introduction:**

Adenylyl cyclase isoform 6 (ADCY6) is a member of membrane-bound adenylate cyclase family that converts adenosine triphosphate (ATP) into cAMP and pyrophosphate. An increasing number of researchers have studied the role of ADCY6 in cancer. However, its specific role in breast cancer remains unknown.

**Methods:**

Bioinformatics and clinical data were used to analyse the expression of ADCY6 in breast cancer. ADCY6 DNA methylation was analysed using DNA methylation-specific PCR and Bisulfite Sanger sequencing. Using lentiviral stable miRNA transfection together with cell biology functional assays and gene expression/target analysis, we investigated the interaction between miR-27a-3p, TET1 and ADCY6 in breast cancer.

**Results:**

We found that ADCY6 is expressed at low levels in breast cancer and leads to increases in the proliferation, invasion and migration of breast cancer cells. The low expression of ADCY6 is due to the lower demethylation of ten-eleven translocation methylcytosine dioxygenase 1 (TET1), and the methylation of ADCY6 can be altered by TET1. More importantly, bioinformatics analysis showed that TET1 is regulated by miR-27a-3p and regulates the methylation of ADCY6 to affect the EMT process of breast cancer cells, thereby affecting the malignant biological behaviour of breast cancer.

**Conclusions:**

Our study demonstrates that the methylation modification of ADCY6 is regulated by TET1 and leads to ADCY6 activation. miR-27a-3p negatively regulates the expression of TET1 and affects the EMT process of breast cancer through ADCY6, thereby promoting the malignant biological behaviour of breast cancer. Our results may provide new research ideas and directions for DNA methylation and EMT changes in breast cancer.

## Introduction

Breast cancer (BCa) is a malignant tumor of breast epithelial tissue and is the most common reproductive system tumor in women (1). BCa has become the most prevalent malignant tumor for women in the world, seriously affecting women’s quality of life and threatening women’s health. Although the incidence of breast cancer is very high, because the breast anatomy is located on the body surface, the early symptoms are more obvious. Coupled with the large-scale popularization of early screening, most breast cancers are diagnosed at an early stage (2). If the treatment is timely, most patients can obtain a better prognosis than other malignant tumors. According to statistics from the American Cancer Federation, the five-year survival rate for breast cancer reaches 91%, and the 10-year survival rate is 84%, which is significantly higher than other tumors.^3^ Most breast cancer patients can maintain long-term survival. However, in the malignant tumor prevalence announced by the National Cancer Center in China in 2015, there were approximately 304,000 new female breast cancer cases, an increase of 11.5% from the previously estimated 269,000 cases (3). The number of breast cancer patients in China is increasing, and the situation is grim. Once a breast cancer patient develops distant metastasis, the patient’s survival time is significantly reduced. The five-year overall survival rate for localized disease is 99%, for local metastasis patients is 86%, and for patients with distant metastasis only 27% (4). It is estimated that 20–30% of early breast cancer patients will eventually develop distant metastases (4). Therefore, research on breast cancer metastasis and recurrence is necessary.

DNA demethylation refers to the lack and reduction of the DNA methyltransferase content in mammalian cells, which is important for turning on the expression of specific genes and the initiation of reprogramming effects (5). The 5-carbon DNA cytosine can lose methylation due to the formation of 5-hydroxymethylcytosine (5’-hmC). 5-hmC is also involved in gene expression and regulation and is related to diseases such as cancer (6). In 1952, Wyatt first discovered that 5’-hmC was the hydroxylated form of 5-methylcytosine (5mC) (7). In 1972, Penn et al. also found hydroxylated modified cytosines in DNA extracted from brain tissues of rats, mice, and oviparous bullfrogs, which accounted for approximately 15% of the total cytosine of DNA (8). TET1, a member of the TET protein family, is an α-ketoglutarate and Fe2+-dependent dioxygenase that converts 5-methylcytosine (5m C) into 5-hydroxymethylcytosine (5hm C), thereby initiating the DNA demethylation program (9, 10). It was found that the down-regulation of TET1 led to hypermethylation of DKK-1 promoter, activation of Wnt signaling pathway, and thus promoted the expression of Fas ligand (FasL), which led to the enhancement of immune regulation ability of periodontal ligament stem cells (11). In breast cancer, FECR1 circular RNA binds to the FLI1 promoter in cis and recruits TET1, reducing the DNA methylation of oncogenes, promoting their gene expression, and driving tumor metastasis through epigenetic mechanisms (12). In addition, TET1 mediated demethylation on CpG island increases the expression of endogenous LRIG1 in basal/triple negative breast cancer cells, and changes the malignant biological behavior in breast cancer cells through epigenetic mechanisms (13). These findings suggest that TET1 plays an important role in regulating DNA demethylation and the malignant progression of breast cancer. Therefore, we focused on the regulation of TET1 on DNA demethylation and protein expression of breast cancer tumor suppressor genes.

Adenylyl cyclase (AC) is a membrane integrin that can convert ATP into cAMP, causing a cell signal response, and is an effector in G protein coupling system (14). AC is widely distributed in mammalian cell membrane. Studies have shown that different subtypes of AC have different connections with various diseases, and there is nonspecific cross-coordination or antagonism between the effects of each subtype (15, 16). Currently, many studies have focused on demonstrating the abnormal expression of ADCY6 in various cancers, including breast cancer (17, 18). Adrenaline can increase the level of circHIPK3 in heart *via* transcription factor CREB1. Furthermore, circ-HIPK3 can affect the concentration of miR-17-3p in cytoplasm through ADCY6 and promote the occurrence of and death by heart failure (19). In osteoporosis, miR-182-5p promotes osteoblast proliferation and differentiation by upregulating ADCY6 and activating the Rap1/MAPK signalling pathway (20). More importantly, high ADCY6 expression and low methylation in breast cancer patients is correlated with better prognosis, and the activation of signalling pathways, immune checkpoint receptors and ligands related to the immune process is negatively correlated (21). In addition, ADCY6 is involved in DNA methylation and immune processes regulated by basalization (21).

In this study, the roles of ADCY6 in breast cancer were systematically investigated, and the mechanism underlying these effects was also explored. This study demonstrates that ADCY6 functions as a tumor suppressor gene in breast cancer. Specifically, the low expression of ADCY6 is due to the low demethylation of TET1. Importantly, the low expression of ADCY6 may regulate malignant biological behavior *via* EMT process.

## Materials and methods

### Patient sample collection

We collected 56 samples from patients with breast cancer (tumor tissues and paired normal adjacent tissues). Breast cancer samples were retrospectively obtained from Department of Breast Surgery, West China Hospital of Sichuan University. The primary cancer tissues and the paired normal adjacent tissues were immediately snap-frozen in liquid nitrogen and stored at 80°C until use for mRNA and protein assessment. All patients provided written informed consent. This study was approved by the Institutional Review Board of West China Hospital of Sichuan University, and written consent was obtained from all participants.

### Cell culture and transfection

SKBR3, MDA-MB-231, MCF-7 and BT-474 were obtained from the Institute of Biochemistry and Cell Biology (Chinese Academy of Sciences, Shanghai, China). MCF-10 cells were obtained from the American Type Culture Collection. SKBR3, MDA-MB-231, MCF-7 and BT-474 cells were cultured in DMEM containing 10% FBS (Corning). MCF-10 cells were cultured in MEBM (CC-3151, Lonza, Basel, Switzerland) supplemented with MEGM^®^ SingleQouts^®^ (CC-4136, Lonza) and 100 ng/mL cholera toxin. Molecular typing of breast cancer cell lines in **Supplementary Table 1**.

Cells were transfected with the following plasmids: shRNA overexpression ADCY6 (Lv-ADCY6), shRNA downregulation ADCY6 (De-ADCY6), shRNA overexpression TET1 (Lv-TET1), shRNA downregulation TET1 (De-TET1), miR-27a-3p mimics (miR-27a-3p), miR-27a-3p inhibitor (De-miR-27a-3p) (Gene Pharma, Shanghai, China). Plasmids were transfected into breast cancer cells with Lipofectamine^®^ 3000 (Invitrogen).

### Immunohistochemistry

Briefly, the samples were dehydrated with xylene and alcohol. Antigen retrieval was performed in 10 mmol/L sodium citrate solution (pH 6.0) at 100°C for 16 min, and the samples were cooled for 30 min. Endogenous peroxidase activity using 3% hydrogen peroxide and blocked with foetal bovine serum for 30 min. Thereafter, the slides were incubated with anti-ADCY6 rabbit polyclonal antibodies (ab14781, 1:500, Abcam, USA), anti-N-cadherin (ab76011, 1:100, Abcam, USA), anti-vimentin (ab92547, 1:200, Abcam, USA), anti-Ki-67(ab16667, 1:200,Abcam, USA), anti-E-cadherin(ab40772, 1:500, Abcam, USA) and anti-twist1(ab175430,1:200, Abcam, USA) at 4°C overnight. Then, the samples incubated with biotinylated secondary antibody at 37°C for 30 min. The samples were stained with DAB (3, 3- diaminobenzidine) and Mayer’s haematoxylin.

ADCY6 staining classifications were as followings: range 0–3: 0, negative; 1, weak; 2, moderate; and 3, strong, the percentage of positive cells range 0–4: 0, negative or <5%; 1, 6%–25%; 2, 26%–50%; 3, 51%–75%; and 4, 76%–100%. Used the percentage of positive cells and the intensity to determine the final staining scores. Grades <4 were defined as low ADCY6 expression, while Grades ≥4 were defined as high ADCY6 expression.

### Western blot

Total protein was isolated from breast cancer tumor tissues, paired normal adjacent tissues and cell lines by protein extraction buffer (RIPA lysis buffer). Total protein was separated on 10% SDS–PAGE gels. The membranes for 1 hour at room temperature with 5% nonfat powdered milk. The membranes were probed at 4°C overnight with anti-ADCY6 (ab14781, 1:3000, Abcam, USA), anti-TET1 (ab191698, 1:2000, Abcam, USA), anti-N-cadherin (ab76011, 1:5,000, Abcam, USA), anti-vimentin (ab92547, 1:2,000, Abcam, USA), anti-Ki-67(ab16667, 1:5,000, Abcam, USA), anti-E-cadherin(ab40772, 1:10,000, Abcam, USA), anti-twist1(ab175430, 1:1,000, Abcam, USA) and anti-β-actin (ab8226, 1:5000, Abcam, USA). Then, the membranes incubated with the appropriate secondary antibodies. Finally, protein expression was analysed by chemiluminescence reagents (Hyperfilm ECL,USA).

### Quantitative real-time PCR (qRT–PCR)

Total RNA was extracted from breast cancer tumor tissues, paired normal adjacent tissues and cell lines by TRIzol reagent (TaKaRa, Japan). The extracted RNA was reverse transcribed to cDNA by PrimeScript RT Reagent (TaKaRa, Japan). qRT–PCR was performed with Prime-Script^®^ RT Reagent Kit (TaKaRa, Japan), and a LightCycler system (Roche) was used for detection. U6 was used as an internal control for miRNA, GAPDH was used for mRNA. Relative RNA expression levels were calculated by the 2^−ΔΔCt^ method. miR-27a-3p forward: 5’ – ATG GTT CGT GGG TTC ACA -3’; reverse: 5’- GTG GCT AAG TTC CGA CG -3’; ADCY6 forward: 5’ - CAG CAG GGT AGT GTG TGC AG-3’; reverse: 5’-TCT GCA TTT GAT TTT GGC CT-3’; TET1 forward: 5’ – TGA TGA CAG AGG TTC TTG CAC ATA AG -3’; reverse: 5’- CAG GTT GCA CGG TCT CAG TGT T 3’; GAPDH: forward: 5’- GGA GCG ACA TCC GTC CAA AAT -3’; reverse: 5’- GGC TGT TGT CAA TCT TCT CAT GG -3’; U6: forward: 5’- CTC GCT TCG GCA GCA CA -3’; reverse: 5’- AAC GCT TCA CGA ATT TGC GT -3’.

### Cell immunofluorescence

Cells were fixed with 4% paraformaldehyde and permeabilized with 0.25% Triton X-100 solution for 20 min. Cells were washed with PBS and then blocked in 5% bovine serum albumin for 1 h at room temperature. Coverslips were incubated with anti-ADCY6 (1:300, Abcam, USA) and anti- TET1 (1:500, Abcam, USA) antibodies overnight at 4°C. After washing with PBS, cells were incubated with appropriate secondary antibody and 4′,6-diamidino-2-phenylindole (DAPI). Slides were imaged using an inverted fluorescence microscope.

### DNA extraction and methylation-specific PCR

Genomic DNA was isolated from breast cancer tumor tissues, paired normal adjacent tissues, and breast cancer cells using a DNA Isolation kit (Tiangen, Beijing, China) according to the manufacturer’s protocol. Determination of bisulfite conversion was performed using the EpiTect Bisulfite Kit (Qiagen). Methylation-specific PCR (MSP) was performed with 2 μL of bisulfite-modified DNA (100 ng/50 μl) and 48 μL of PCR mixture consisting of 10 × PCR Buffer (Mg2+ free), 25 mM MgCl2, dNTP mixture (each 2.5 mM), sense primer (20 μM), antisense primer (20 μM), and TaKaRa EpiTaq HS (5 U/μL; TaKaRa). PCR amplification was conducted using 40 cycles (96°C for 15 s, 50°C for 30 s, and 72°C for 30 s). For parallel quality control, a plasmid containing a methylated ADCY6 sequence and water without DNA template were used as positive and negative controls, respectively.

### TCGA and CPTAC analysis

We used UALCAN (http://ualcan.path.uab.edu/cgi-bin/ualcan-res.pl) to analyse the expression of ADCY6 in the TCGA and CPTAC databases.

### Bisulfite Sanger sequencing (BSP)

At least 500 ng of genomic DNA extracted from breast cancer patient specimens and breast cancer cell lines was bisulfite converted using a MethylCode™ Bisulfite Conversion Kit (Applied Biosystems, USA). The ADCY6 promoter was amplified by PCR with Taq DNA Polymerase (Invitrogen, USA). The primer sequence was designed using Methyl Primer Express™ Software v1.0 (Applied Biosystems, USA). The PCR products were electrophoresed, purified using Spin−X tubes, and then cloned into the pUC-T vector (both from CWBiotech, Beijing, China). Ten single products were sequenced for each sample.

### miRNA prediction

Three prediction databases, TargetScan (http://www.targetscan.org), Oncomir (http://www.oncomir.org/), and miRWalk (http://mirwalk.umm.uni-heidelberg.de/), were used to predict miRNA targets and conserved sites bound by TET1.

### Dual luciferase reporter assay

TargetScan (http://www.targetscan.org) was used to identify downstream target genes of miR-27a-3p. The wild-type (WT) TET1-3’UTR and mutant (MUT) TET1-3’UTR oligonucleotides containing the putative binding site of miR-27a-3p were cloned into the firefly luciferase-expressing pMIR-REPORT vector (Obio Technology Corp., Ltd.). These constructs were cotransfected with miR-27a-3p mimics, miRNA negative control mimics, miR-27a-3p inhibitor, miRNA-NC inhibitor into breast cancer cells. After 48 h of transfection, luciferase activity was determined using the Dual-Luciferase Reporter Assay kit (Promega Corporation) according to the manufacturer’s protocol. The ratio of Renilla luciferase activity to firefly luciferase activity was calculated.

### Cell-counting Kit-8 assay

Cells were transfected and then inoculated into 96-well plates. CCK8 assay reagent to 96-well plates inoculated with transfected cells according to the manufacturer’s instructions. The absorbance at 450 nm was recorded for each well to assess cell proliferation.

### 5-Ethynyl-2’-deoxyuridine (EdU) assay

Cells were transfected and inoculated into 96-well plates. Then, the cells were immobilized with 4% polyformaldehyde, and nuclei were with 0.5% Triton X-100 solution. EdU (50 μM), 1× ApolloR reaction cocktail (100 μl) and 1× DAPI (100 μl) to 96-well plates. Cell proliferation was analysed using the mean number cells each group.

### Colony-forming assay

Cells were transfected and inoculated into in 6-well plates (500 cells/well) containing 2.5 mL of medium and cultured for two weeks. Colonies formed by cell proliferation were fixed with 4% paraformaldehyde and stained by 0.1% crystal violet, and the colonies were counted.

### Cell migration and invasion assays

Cell invasion and migration assays were performed using Transwell chambers (Corning Costar, Cambridge, MA, USA). MDA-MB-231 and MCF-7 cells were seeded into the upper chamber of the Transwell chambers. Then, 500 µl of high-glucose DMEM containing 10% FBS was added to the matched lower chamber. After incubation for 48 h, MDA-MB-231 and MCF-7 cells moved to the lower chamber. The cells were fixed in methanol and stained with 0.1% crystal violet. For the invasion assay, the inserts were precoated with Matrigel (1 mg/mL).

### Mouse xenograft tumor model

Five-week-old female BALB/c-nu mice were purchased from Shanghai Experimental Animal Center (Shanghai, China) and fed in the Experimental Animal Center of West China Hospital of Sichuan University. All animal experiments were performed in accordance with institutional guidelines. For the *in vivo* cell proliferation study, before and after treatment MDA-MB-231 and MCF-7 (5x10^6^) were subcutaneously injected into the mammary fat pads of female athymic nude mice. Tumor growth was recorded once a week with calliper measurements. Tumor volume was calculated according to the following formula: volume = (width^2^ × length)/2. At 40 days after injection, the mice were euthanized, and tumors were collected for analysis.

### Statistical analysis

Statistical differences were analysed by Student’s t test and Pearson correlation coefficient was used for comparisons. For multiple group comparisons, ANOVA (analysis of variance) was performed. Tukey’s test was used as the *post hoc* test after ANOVA. The correlation between ADCY6 and TET1 mRNA expression was evaluated using Spearman’s correlation analysis. The threshold for statistical significance was p < 0.05. All data were analysed using SPSS 22.0 software (SPSS Inc., Chicago, IL, USA) or GraphPad Prism version 7.0 (CA, USA). Data sre presented as means ± standard deviations (SD).

## Results

### Differential expression of ADCY6 in breast cancer tissues and cells

**Figure 1:**
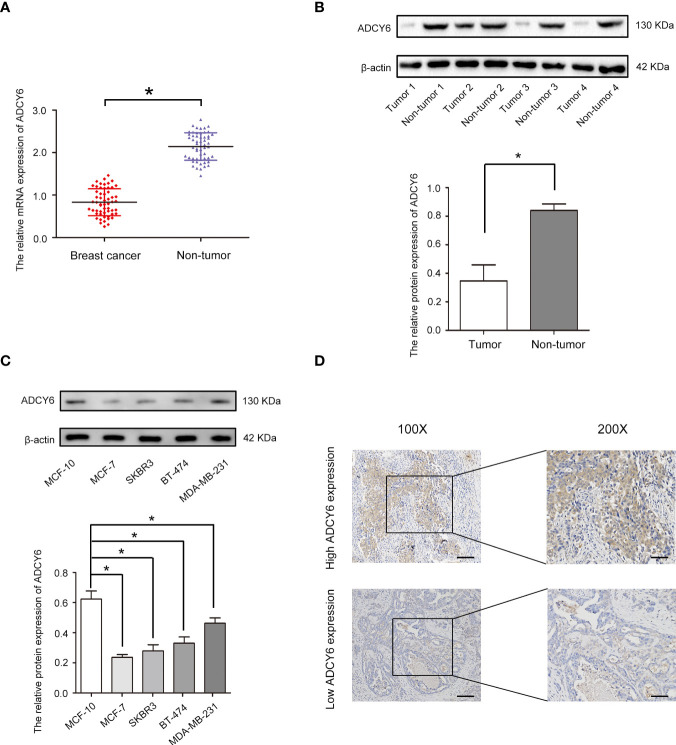
ADCY6 expression is decreased in breast cancer samples and cell lines. **(A)** The mRNA expression of ADCY6 in breast cancer samples. N = 56. *p < 0.05 versus the adjacent normal tissues. **(B)** The protein expression of ADCY6 in breast cancer samples. *p < 0.05 versus the adjacent normal tissues. **(C)** The protein expression of ADCY6 in breast cancer cell lines. *p < 0.05 versus MCF-10 cells. **(D)** ADCY6 staining was performed in representative breast cancer samples. Scale bars, 100 mm.

We analysed the expression of ADCY6 through the TCGA and CPTAC databases (**Supplementary Figure S1**). Based on the above analysis results, we detected the expression of ADCY6 in breast cancer patient samples collected by ourselves. qRT–PCR results showed that ADCY6 was expressed at low levels in breast cancer tissues (**Figure 1A**). In addition, WB results also confirmed the protein expression of ADCY6 was lower in breast cancer tissues than in paired normal adjacent tissues (**Figure 1B**). Unsurprisingly, the expression of ADCY6 was also lower in breast cancer cells (**Figure 1C**). Immunoblotting suggested that ADCY6 was expressed at low levels in breast cancer tissues, and mainly expressed in the cytoplasm of breast cancer cells (**Figure 1D**). In addition, ADCY6 expression was positively correlated with lymph node status (P = 0.027) and clinical stages (P = 0.034) (**Table 1**).

**Table 1:** Association of ADCY6 expression with clinicopathological characteristics in Breast Cancer.

Variables	Cases	ADCY6 expression		*P*
		Low (n=42)	High (n=14)	
Lymph Node status				0.027
Positive	34	29	5	
Negative	22	13	9	
Clinical stages				0.034
Grade 1 and 2	19	11	8	
Grade 3 and 4	37	31	6	
Tumour histological subtypes				0.417
Ductal NST	51	39	12	
Lobular Medullary like Special type	0 5 0	0 3 0	0 2 0	
Oestrogen receptor (ER)				0.871
Positive	37	28	9	
Negative	19	14	5	
Progesterone receptor (PR)				0.142
Positive	37	30	7	
Negative	19	12	7	
Human epidermal growth factor receptor 2 (HER2)				0.867
Positive	17	13	4	
Negative	39	29	10	
Molecular subtypes				0.909
Luminal A	41	31	10	
Luminal B HER-enriched Basal like Normal like	9 4 1 1	6 3 1 1	3 1 0 0	

### Increase ADCY6 expression significantly inhibited the proliferation of breast cancer cells

**Figure 2:**
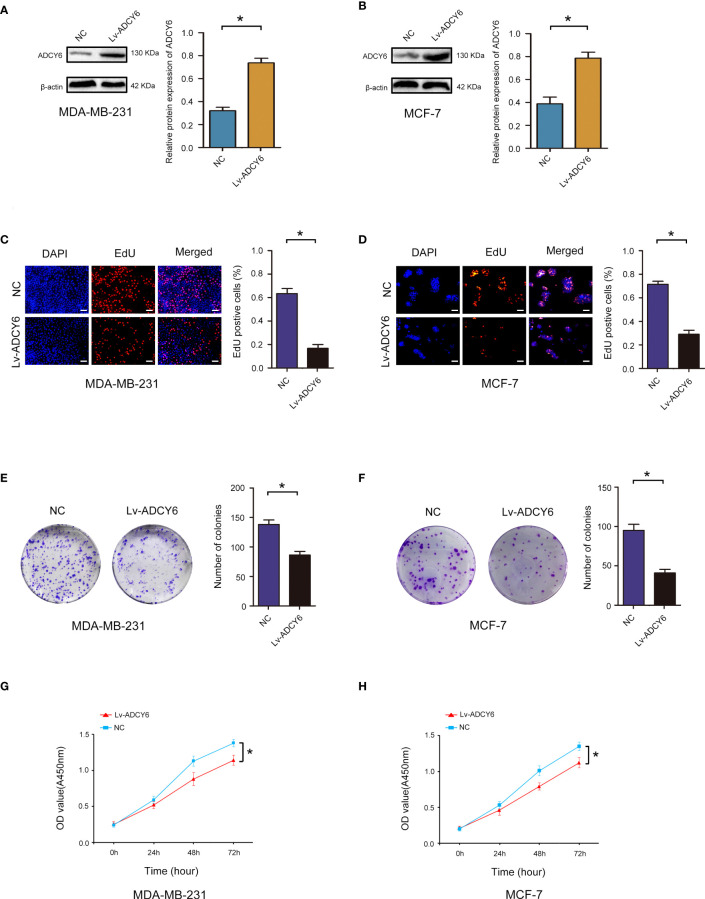
ADCY6 significantly suppressed the proliferation of breast cancer cell lines. **(A)** Western blot assay showing the protein expression of ADCY6 in ADCY6 increased (Lv-ADCY6) MD-MBA-231 cells. *p < 0.05 versus controls. **(B)** Western blot assay showing the protein expression of ADCY6 in ADCY6 increased (Lv-ADCY6) MCF-7 cells. *p < 0.05 versus controls. **(C)** EdU assay to detect the uptake of EdU in MD-MBA-231 cells. Scale bars, 50 µm. *p < 0.05 versus controls. **(D)** EdU assay to detect the uptake of EdU in MCF-7 cells. Scale bars, 50 µm. *p < 0.05 versus controls. **(E)** Colony formation assay to detect proliferation in MD-MBA-231cells. *p < 0.05 versus controls. **(F)** Colony formation assay to detect the proliferation of MCF-7 cells. *p < 0.05 versus controls. **(G)** CCK-8 assay to detect the proliferation of MD-MBA-231 cells. *p < 0.05 versus controls. **(H)** CCK-8 assay to detect the proliferation of MCF-7 cells. *p < 0.05 versus controls.

To assess the roles of ADCY6 in breast cancer, gain-of-function methods were used. We generated MDA-MB-231 and MCF-7 cells with increased ADCY6 expression (Lv-ADCY6). Western blotting was performed to verify the protein levels of ADCY6 after transfection. As shown in **Figures 2A, B**, ADCY6 protein levels were increased in Lv-ADCY6-MDA-MB-231 and Lv-ADCY6-MCF-7 cells. Increasing the expression of ADCY6 significantly inhibited the uptake of EdU by MDA-MB-231 and MCF-7 (**Figures 2C, D**). Cell cloning experiments showed that increasing the expression of ADCY6 markably inhibited the growth of MDA-MB-231 and MCF-7 cells (**Figures 2E, F**). Subsequently, CCK-8 results showed that increasing the expression of ADCY6 significantly inhibited cell proliferation (**Figures 2G, H**). Hence, increasing the expression of ADCY6 could inhibit proliferation of breast cancer cells.

### Increase ADCY6 expression significantly inhibited breast cancer cell invasion and migration

**Figure 3:**
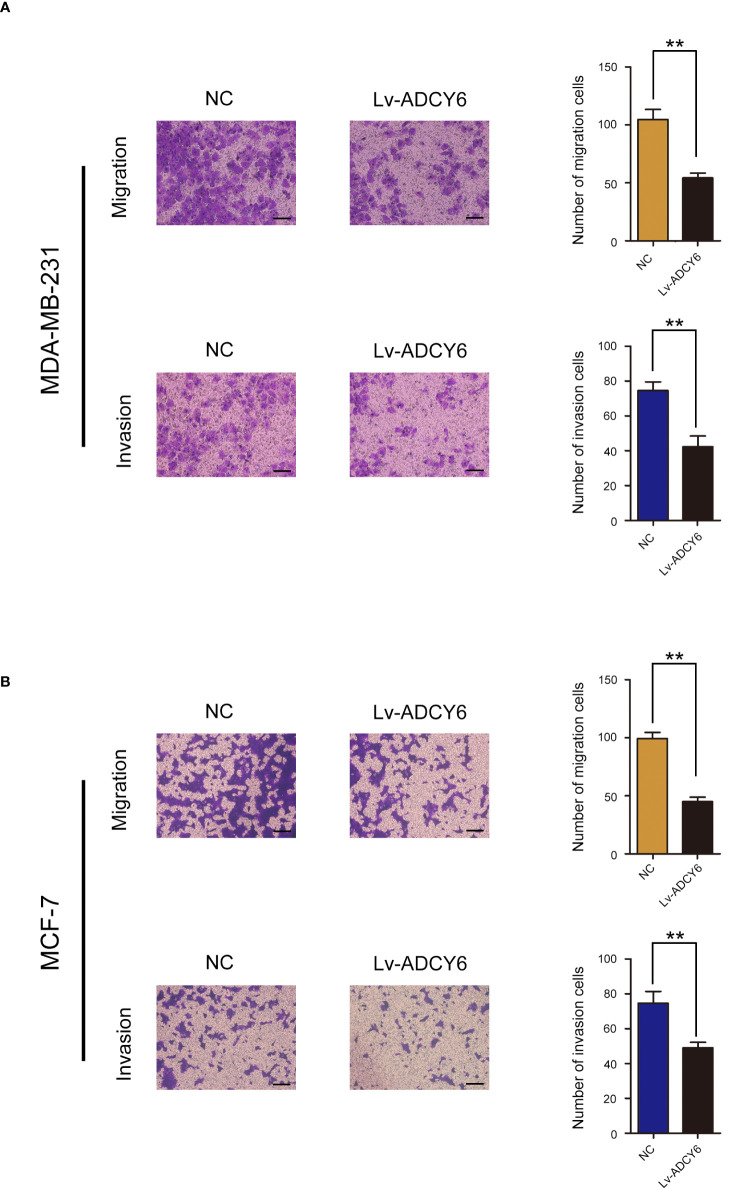
ADCY6 significantly suppressed the migration and invasion of breast cancer cell lines. **(A)** Transwell assay to detect MD-MBA-231 cell migration and invasion. Scale bars, 100 µm. **p < 0.01 versus controls. **(B)** Transwell assay to detect MCF-7 cell migration and invasion. Scale bars, 100 µm. **p < 0.01 versus controls.

Furthermore, the effects of ADCY6 on invasion and migration of breast cancer cells were evaluated by Transwell assays. Transwell results showed that increasing the expression of ADCY6 significantly inhibited the migration and invasion of MDA-MB-231 and MCF-7 (**Figures 3A, B**).

### Methylation of ADCY6 promoter CpG islands contributes to ADCY6 downregulation in BCa

**Figure 4:**
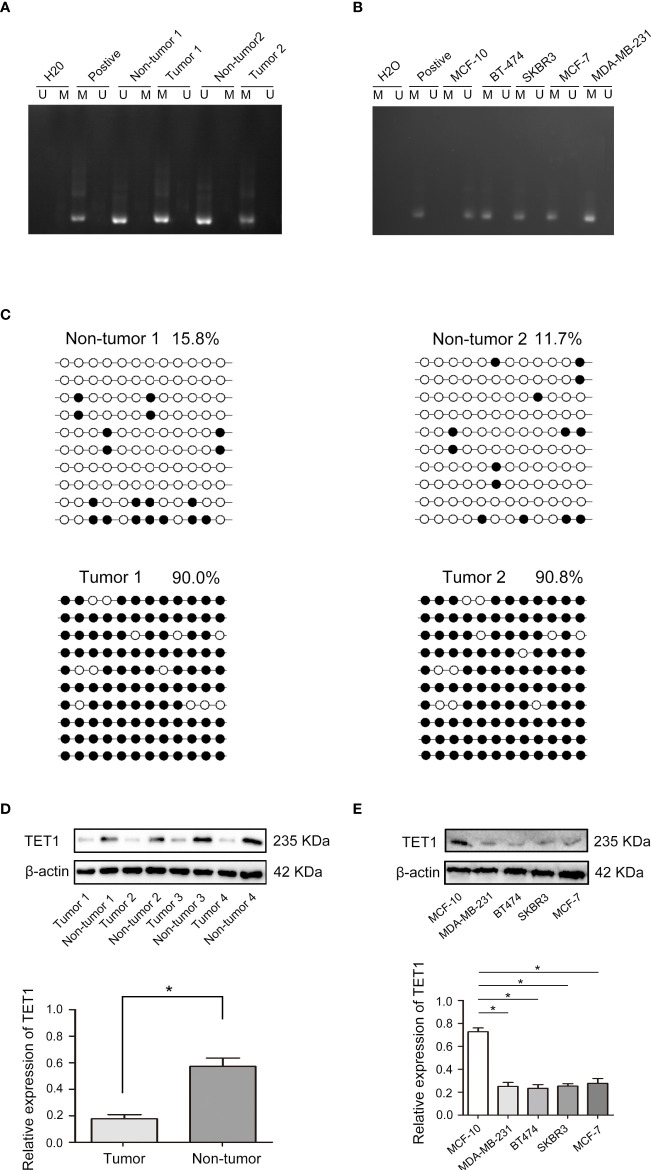
Aberrant DNA hypermethylation of ADCY6 and aberrant expression of TET1 in breast cancer. **(A)** The methylation status of ADCY6 was randomly detected in breast cancer tissues. **(B)** The methylation status of ADCY6 was detected in breast cancer cell lines. **(C)** Bisulfite sequencing analysis was performed on ADCY6 promoter methylation in randomly detected breast cancer tissues. Black dots, methylated; white dots, unmethylated. **(D)** The protein expression of TET1 in breast cancer samples. *p < 0.05 versus adjacent normal tissues. **(E)** The protein expression of TET1 in breast cancer cell lines. *p < 0.05 versus MCF-10 cells. Black dots and M, methylated; white dots and U, unmethylated.

In breast cancer, ADCY6 has been reported to aberrantly regulate DNA methylation, leading to the malignant growth of breast cancer (21). The methylation status of ADCY6 was assayed in randomly selected BCa tissues compared with paired adjacent normal tissues. The results showed that the methylation of ADCY6 was high in breast cancer (**Figure 4A**). In addition, analysis of MCF-10, BT-474, SKBR3, MCF-7, and MDA-MB-231 revealed a significantly high degree of methylation in BCa cell line, but not in MCF-10 cells (**Figure 4B**). Next, we assessed the exact methylation of ADCY6 by BSP. ADCY6 promoter methylation in BCa tissues was increased compared with paired adjacent normal tissues (**Figure 4C**). DNA methylation is a dynamic regulatory process of methyltransferases and demethylases (22). To determine whether the hypomethylation of ADCY6 caused by demethylase dysfunction, we detected the expression of key enzyme TET1, which activates DNA demethylation. As shown in **Figure 4D**, the expression of TET1 in BCa tumor tissues was downregulated compared with paired adjacent normal tissues. The same results were also observed in BCa cells (**Figure 4E**). These results strongly suggest that ADCY6 undergoes demethylation in BCa, which may be caused by TET1.

### TET1 increased the expression of ADCY6 through demethylation

**Figure 5:**
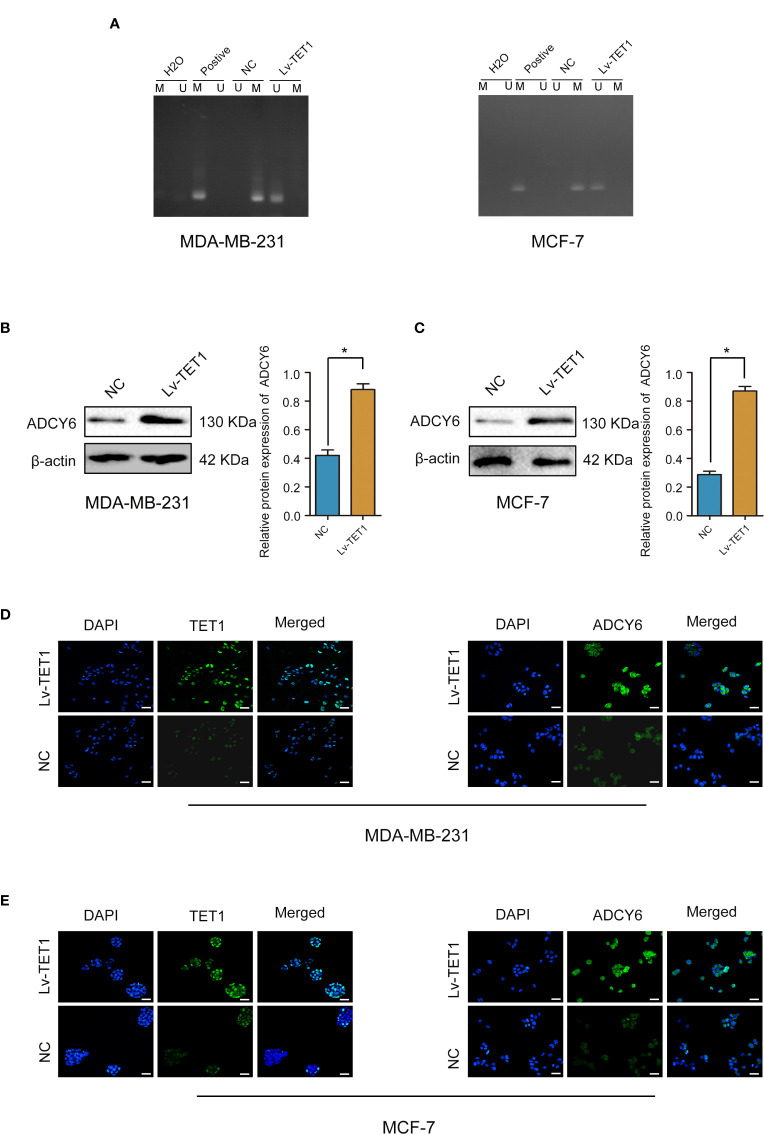
TET1 expression was positively correlated with ADCY6 protein expression. **(A)** The methylation status of ADCY6 after increasing the expression of TET1 in breast cancer cell lines. **(B)** The protein expression of ADCY6 after increasing the expression of TET1 was detected by western blot in MDA-MB-231 cells. *p < 0.05 versus controls. **(C)** The protein expression of ADCY6 after increasing the expression of TET1 was detected by western blot in MCF-7 cells. *p < 0.05 versus controls. **(D)** The protein expression of TET1 and ADCY6 after increasing the expression of TET1 was detected by immunofluorescence in MDA-MB-231 cells. Scale bars, 50 µm. **(E)** The protein expression of TET1 and ADCY6 after increasing the expression of TET1 were detected by immunofluorescence in MCF-7 cells. Scale bars, 50 µm.

Our results demonstrated that increasing the expression of TET1 decreased the methylation of ADCY6 (**Figure 5A**). Next, we effectively increased the expression of TET1, and found that the expression of ADCY6 was significantly increased after increasing the expression of TET1 (**Figures 5B–E**).

### TET1 ameliorates the inhibitory effect of ADCY6 on BCa progression

**Figure 6:**
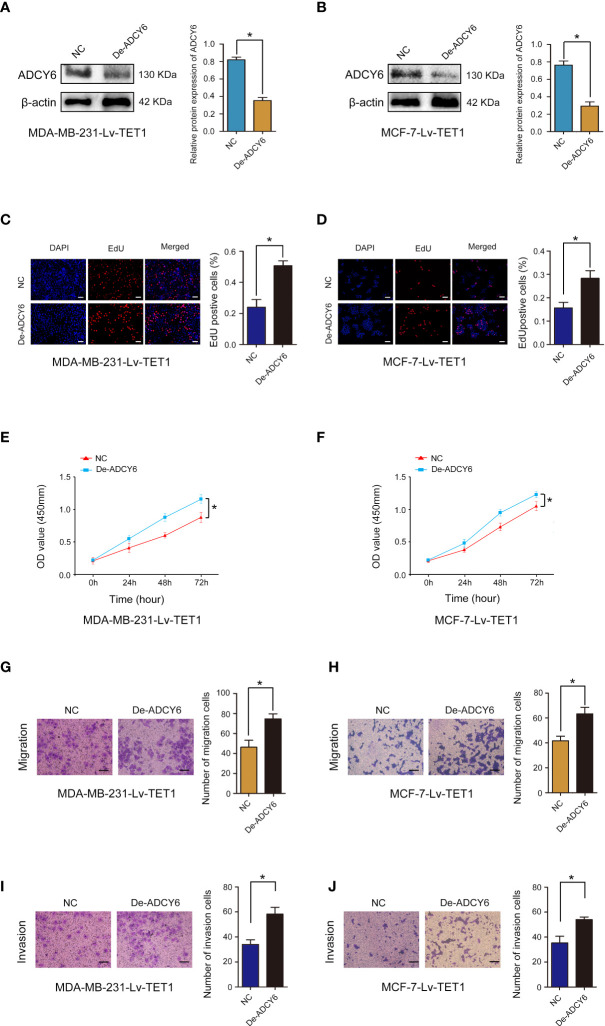
Rescue experiments were performed to confirm that TET1 ameliorates the inhibitory effect of ADCY6 on BCa progression. **(A)** The protein expression of ADCY6 after decreasing ADCY6 expression in MDA-MB-231-Lv-TET1. *p < 0.05 versus controls. **(B)** The protein expression of ADCY6 after decreasing the expression of ADCY6 in MCF-7-Lv-TET1 cells. *p < 0.05 versus controls. **(C)** The uptake capacity of EdU in MDA-MB-231-Lv-TET1 cells that decreased the expression of ADCY6 was determined by EdU assay. Scale bars, 50 µm. *p < 0.05 versus controls. **(D)** The uptake capacity of EdU in MCF-7-Lv-TET1 cells that decreased the expression of ADCY6 was determined by EdU assay. Scale bars, 50 µm. *p < 0.05 versus controls. **(E)** Proliferation of MDA-MB-231-Lv-TET1 cells that decreased the expression of ADCY6 was determined by CCK-8 assay. *p < 0.05 versus controls. **(F)** Proliferation of MCF-7-Lv-TET1 cells that decreased the expression of ADCY6 was determined by CCK-8 assay. *p < 0.05 versus controls. **(G)** The migration of MDA-MB-231-Lv-TET1 cells that decreased the expression of ADCY6 was determined by Transwell assay. *p < 0.05 versus controls. **(H)** The migration of MCF-7-Lv-TET1 cells that decreased the expression of ADCY6 was determined by Transwell assay. *p < 0.05 versus controls. **(I)** The invasion of MDA-MB-231-Lv-TET1 cells that decreased the expression of ADCY6 was determined by Transwell assay. *p < 0.05 versus controls. **(J)** The invasion of MCF-7-Lv-TET1 cells that decreased the expression of ADCY6 was determined by transwell assay. *p < 0.05 versus controls.

Cell function experiments were performed to confirm whether TET1 can regulate the expression of ADCY6 to affect the malignant biological behaviour of BCa. ADCY6 expression was decreased after decreasing the expression of ADCY6 in MDA-MB-231-Lv-TET1 and MCF-7-Lv-TET1 cells (**Figures 6A, B**). Increased of TET1 expression significantly abolished the promotion effects of low expression of ADCY6 on proliferation, migration and invasion in MDA-MB-231-Lv-TET1 and MCF-7-Lv-TET1 cells (**Figures 6C–J**).

### Verification of TET1 as a target gene of miR-27a-3p and TET1 downregulation by miR-27a-3p

**Figure 7:**
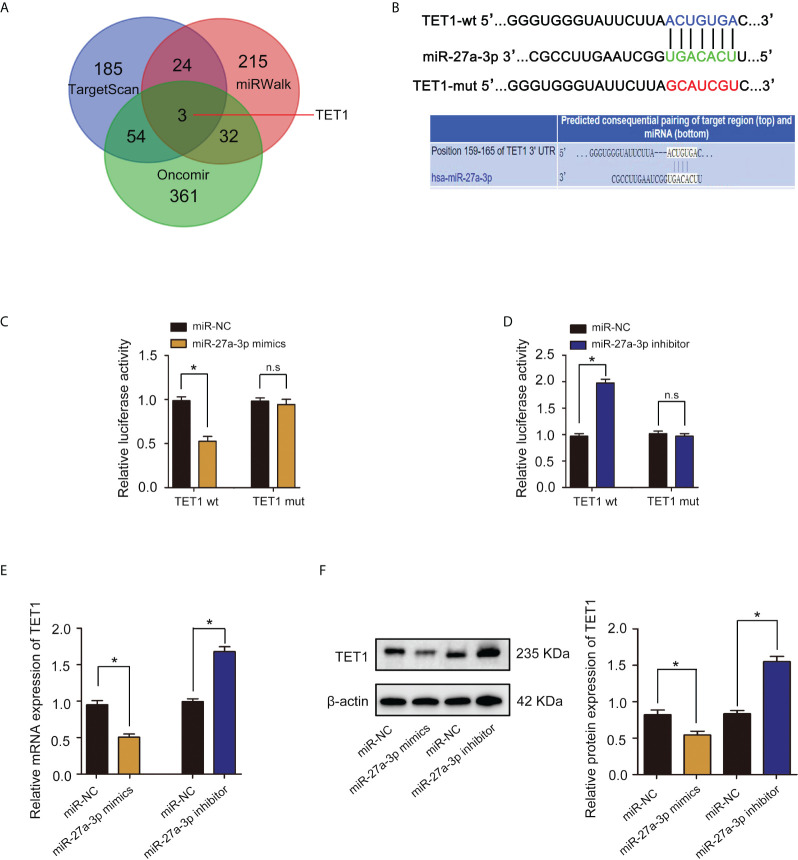
miR-27a-3p directly targets TET1 and downregulates TET1. **(A)** Venn diagram displaying miR-27a-3p computationally predicted to target TET1 by TargetScan, Oncomir, and miRWalk databases. **(B)** The binding sites and corresponding mutant sites of miR-27a-3p and TET1 3’ UTR. **(C)** A dual luciferase activity assay was performed by cotransfection of luciferase reporter containing TET1 3’ UTR or the mutant reporter with miR-27a-3p mimics. *p < 0.05 versus controls. n.s > 0.05 versus controls. **(D)** A dual luciferase activity assay was performed by cotransfection of luciferase reporter containing TET1 3’ UTR or the mutant reporter with miR-27a-3p inhibitor. *p < 0.05 versus controls. n.s > 0.05 versus controls. **(E)** The expression of TET1 detected by qRT–PCR. *p < 0.05 versus controls. **(F)** The protein expression of TET1 detected by western blotting. *p < 0.05 versus controls.

MicroRNA (miRNA) regulation plays important role in methylation modification and tumor progression (23). We examined miRNA databases, including TargetScan, miRWalk and Oncomir and identified TET1 as a potential target of miR-27a-3p (**Figure 7A**). Using bioinformatics website TargetScan for mRNA target analysis, we found that miR-27a-3p contained a potential binding site in TET1 (**Figure 7B**). A dual luciferase reporter gene assay was employed to verify whether TET1 was a target gene of miR-27a-3p. The results showed that cotransfection of miR-27a-3p mimics significantly inhibited luciferase activity in cells cotransfected with Wt TET13’-UTR. In contrast, inhibition was not observed in cells cotransfected with Mut TET1 3’-UTR (**Figure 7C**). In addition, cotransfection of miR-27a-3p inhibitor significantly increased luciferase activity in cells transfected with Wt TET1 3’-UTR. However, the increase was not observed in cells cotransfected with Mut TET1 3’-UTR (**Figure 7D**). qRT–PCR and western blot assays revealed that miR-27a-3p overexpression significantly reduced TET1 mRNA and protein levels (**Figures 7E, F**). These results suggest that miR-27a-3p directly targets and downregulates TET1.

### ADCY6 ameliorates the inhibitory effect of miR-27a-3p on BCa progression

**Figure 8:**
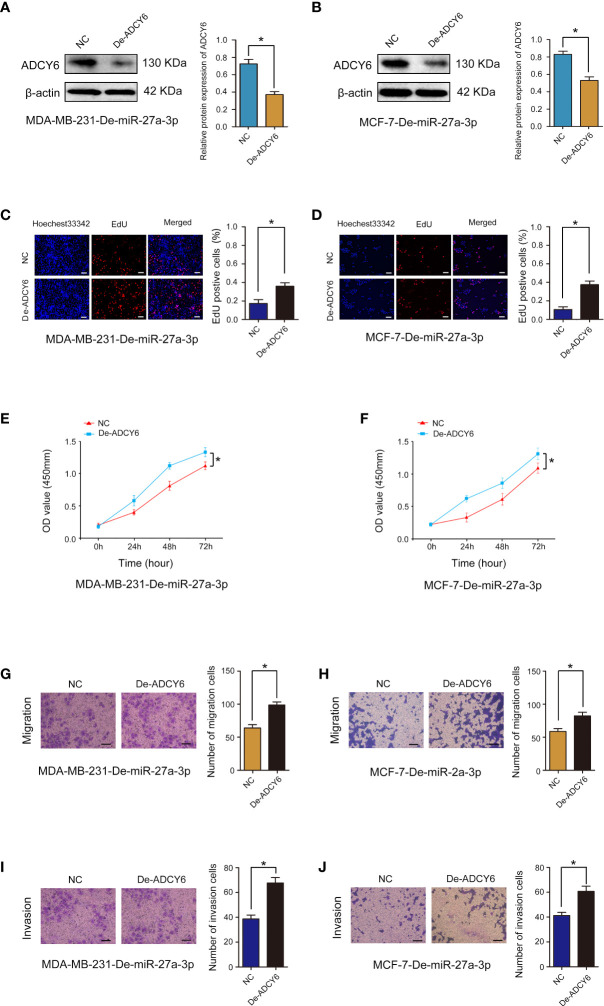
Rescue experiments were performed to confirm that ADCY6 ameliorates the promoting effect of miR-27a-3p on BCa progression. **(A)** The protein expression of ADCY6 after decreasing the expression of ADCY6 in MDA-MB-231-De-miR-27a-3p cells. *p < 0.05 versus controls. **(B)** The protein expression of ADCY6 after decreasing the expression of ADCY6 in MCF-7- De-miR-27a-3p cells. *p < 0.05 versus controls. **(C)** The uptake capacity of EdU in MDA-MB-231-De-miR-27a-3p cells that decreased the expression of ADCY6 was determined by EdU assay. Scale bars, 50 µm. *p < 0.05 versus controls. **(D)** The uptake capacity of EdU in MCF-7-De-miR-27a-3p cells that decreased the expression of ADCY6 was determined by EdU assay. Scale bars, 50 µm. *p < 0.05 versus controls. **(E)** The proliferation of MDA-MB-231-De-miR-27a-3p cells that decreased the expression of ADCY6 was determined by CCK-8 assay. *p < 0.05 versus controls. **(F)** The proliferation of MCF-7-De-miR-27a-3p cells that decreased the expression of ADCY6 was determined by CCK-8 assay. *p < 0.05 versus controls. **(G)** The migration of MDA-MB-231-De-miR-27a-3p cells that decreased the expression of ADCY6 was determined by Transwell assay. *p < 0.05 versus controls. **(H)** The migration of MCF-7-De-miR-27a-3p cells that decreased the expression of ADCY6 was determined by Transwell assay. *p < 0.05 versus controls. **(I)** The invasion of MDA-MB-231-De-miR-27a-3p cells that decreased the expression of ADCY6 was determined by Transwell assay. *p < 0.05 versus controls. **(J)** The invasion of MCF-7-De-miR-27a-3p cells that decreased the expression of ADCY6 was determined by Transwell assay. *p < 0.05 versus controls.

We examined the expression of miR-27a-3p in breast cancer and cell lines, and found that miR-27a-3p was highly expressed in breast cancer and cell lines (**Supplementary Figure S2**). We further confirmed the role of miR-27a-3p and whether miR-27a-3p can regulate the expression of ADCY6 to affect the malignant biological behaviour of BCa. First, miR-27a-3p downregulation markedly suppressed proliferation, migration and invasion in MDA-MB-231 and MCF-7 cells (**Supplementary Figure S3**). In addition, ADCY6 expression was decreased after decreasing the expression of ADCY6 in MDA-MB-231-De-miR-27a-3p and MCF-7-De-miR-27a-3p cells (**Figures 8A, B**). Decreasing the expression of ADCY6 significantly abolished the inhibitory effects of miR-27a-3p on proliferation, migration and invasion in MDA-MB-231 and MCF-7 cells (**Figures 8C–J**).

### Downregulation of miR-27a-3p suppresses tumorigenicity of breast cancer through ADCY6

**Figure 9:**
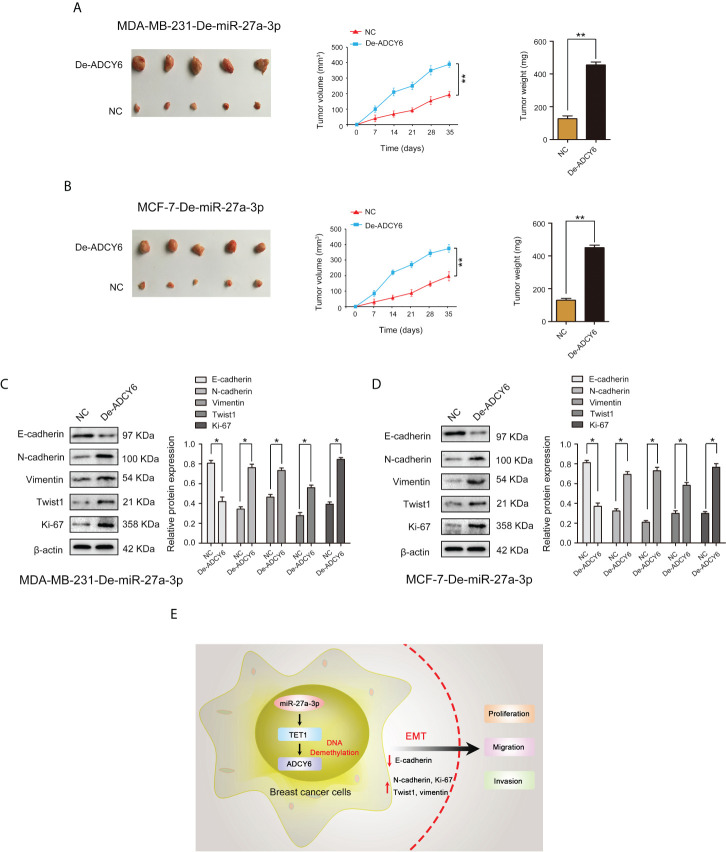
ADCY6 ameliorates the promoting effect of miR-27a-3p on BCa tumorigenicity. **(A)** The tumor volume and weight *in vivo* in each group detected by nude mouse tumor xenograft assay of MDA-MB-231-De-miR-27a-3p cells after decreased the expression of ADCY6. **p < 0.01 versus controls. **(B)** The tumor volume and weight *in vivo* in each group detected by nude mouse tumor xenograft assay of MCF-7-De-miR-27a-3p cells after decreased the expression of ADCY6. **p < 0.01 versus controls. **(C)** Protein expression of EMT components, including: E-cadherin, N-cadherin, vimentin, twist1 and Ki-67 in MDA-MB-231-De-miR-27a-3p cells after decreased the expression of ADCY6. *p < 0.05 versus controls. **(D)** Protein expression of EMT components, including: E-cadherin, N-cadherin, vimentin, twist1 and Ki-67 in MCF-7-De-miR-27a-3p cells after decreased the expression of ADCY6. *p < 0.05 versus controls. **(E)** miR-27a-3p binds to TET1, and downregulates TET1 to activate EMT by promoting DNA methylation of ADCY6, thus promoting proliferation, migration and invasion of breast cancer cells.

Using *in vivo* experiments, we verified whether miR-27a-3p performs biological functions through ADCY6. Subcutaneous ectopic tumors were induced in nude mice to detect the effects of miR-27a-3p and ADCY6 on breast cancer tumor volume and size. The results showed that decrease in ADCY6 expression abolished the inhibitory effects of miR-27a-3p on the tumorigenicity of MDA-MB-231 and MCF-7 cells (**Figures 9A, B**). Based on the results of *in vivo* and *in vitro* experiments, we further analysed whether miR-27a-3p and ADCY6 affect the malignant biological behaviour of breast cancer by regulating EMT. Western blot results revealed that decrease in ADCY6 expression, the expression of N-cadherin, vimentin, twist1 and Ki-67, which were inhibited by downregulation of miR-27a-3p, were restored to a certain extent, and the expression of E-cadherin, which was increased by the downregulation of miR-27a-3p, was inhibited to a certain extent (**Figures 9C, D**). The immunohistochemical results also verified the above results (**Supplementary Figure S4**). In addition, we further confirmed that the miR-27a-3p/TET1 regulatory axis plays an important role in regulating EMT *via* ADCY6 (**Supplementary Figure S5**, **Supplementary Figure S6**). Overall, these results demonstrated that miR-27a-3p binds to TET1 and downregulates TET1 to activate EMT by promoting DNA methylation of ADCY6, thus promoting proliferation, migration and invasion of breast cancer cells (**Figure 9E**).

## Discussion

Breast cancer is the most malignant tumor that endangers women’s lives and health worldwide. It is also the most malignant tumor in China (5). The incidence rate is younger in recent years; the younger the prognosis, the greater the economic and mental burden on society as a whole (5). Increasing evidence shows that epigenetic modifications, especially methylation modifications, play role in the occurrence and development of breast cancer (24). For the first time, this study demonstrates that TET1 increases the expression of ADCY6 through demethylation, and inhibits the progression of malignant biological behaviour of breast cancer by inhibiting EMT signaling pathway.

Epigenetic modification determines how DNA is translated, the strict regulation of DNA structure, and the genes that are expressed at specific time (25). A variety of epigenetic factors coordinate the basic cellular processes from development to cell death pathways (26). Dysfunction of any of these factors disrupts the regulation of the genome, leading to errors in cellular processes, causing cancer and autoimmune disorders, neurological diseases, infertility and other diseases (26). DNA methylation, as an important part of epigenetic modification, was originally found to be located in CpG islands, which are common DNA fragments in gene promoter regions rich in CpG dinucleotides (27). In these promoter regions, DNA methylation acts as a stable epigenetic marker that inhibits gene transcription. In the mammalian genome, methylated cytosine is initially integrated into DNA by *de novo* methyltransferases DNMT3a and DNMT3b during early development (28). Subsequently, an additional methyltransferase, DNMT1, replicates the DNA methylation pattern to the daughter strands during DNA replication, thereby maintaining these methylation markers throughout the genome (28). Numerous studies have shown that DNA methylation inhibits the expression of tumor suppressor genes in tumor cells, thereby losing the normal tumor suppressor effect and promoting the malignant development of tumors (29).

Adenylate cyclase 6 (ADCY6) is a member of membrane-bound adenylate cyclase family (30). ADCY6 converts adenosine triphosphate into cyclic adenosine monophosphate (cAMP) and pyrophosphate (31). cAMP itself is a key regulator of glucose and lipid metabolism (31). Therefore, most of the existing studies focus on the abnormal expression and dysfunction of ADCY6 in different malignant tumor tissues. In NSCLC, overexpression of miR-542-5p significantly inhibited tumor growth and angiogenesis, and its downstream target genes mostly came from cAMP signalling pathway, among which ADCY6 was an important target gene and was shown to be overexpressed in NSCLC tissues and participate in the process of miR-542-5p regulating tumor growth (32). Through bioinformatics analysis, the target gene was found to play a regulatory role in laryngeal squamous cell carcinoma tumor tissue. The abnormal expression of ADCY6 has been confirmed, and it is involved in processes such as apoptosis, cell cycle, DNA repair, protease inhibition, signal transduction and transcriptional regulation (33). In luminal-like breast cancer, ADCY6 is modified by DNA methylation, and patients with low methylation and high expression have a better prognosis (21). Meanwhile, the expression level of ADCY6 is negatively correlated with the activation of immune signalling pathways, immune checkpoint receptors and ligands (21). These results suggest that ADCY6, as an important prognostic factor, participates in the immune process of DNA methylation regulation. Our results were consistent with previous studies. We found that the low level of ADCY6 protein expression was caused by its high methylation level. Increasing the expression of TET1 could inhibit the malignant biological behaviour of breast cancer cells and suggests that the high methylation level of ADCY6 is due to the low TET1 expression in breast cancer.

DNA methylation is a dynamic process that includes DNA methylation and demethylation (34). The process of DNA demethylation eventually removes 5-methylcytosine (5mC), and converts methylated cytosine into unmodified cytosine (C) (35). Active DNA demethylation is one of the main routes of DNA demethylation, and it starts at 5mC and culminates in unmodified C (36). TET1 can catalyse the conversion of 5-methylcytosine (5-mc) to 5-hydroxymethylcytosine (5-hmc), which is an important enzyme in the process of DNA demethylation (37). Previous studies have found that TET1 plays an increasingly important role in the malignant progression of tumors (38, 39). Transcriptome analysis of liver cancer samples indicated that TET1 was highly expressed in liver cancer tissues, and TET1 gene knockout in liver cancer cell lines reduced hmC deposition and inhibited cell growth. Meanwhile, TET1 promotes cell proliferation by regulating t oncogenic target HMGA2 (40). In addition, CD147 gene has undergone active demethylation in NSCLC, which upregulates the expression of CD147 (41). TET1 can significantly regulate the methylation and protein expression of CD147 through demethylation (41). In triple-negative breast cancer cells, EZH2 reduced TET1 expression through epigenetic regulation of H3K27me3, thereby inhibiting the antitumor p53 signalling pathway. Analysis of clinical data shows that patients with high EZH2 and low TET1 have the worst survival outcomes (42). Our research demonstrates that TET1 is expressed at low levels in breast cancer, leading to the low demethylation of ADCY6, inhibiting ADCY6 protein expression, and regulating the malignant biological behaviour of breast cancer through EMT signalling pathway. DNA methylation is a dynamic and reversible process. DNMT3b, as a key enzyme of methyl transfer, can regulate the DNA methylation level of LATS1 and affect the malignant biological behaviour of HCC through Hippo signalling pathway (43). In addition, MUC1-C induces the expression of DNMT1 and DNMT3b in breast cancer and induces the DNA methylation of CDH1 tumor suppressor gene and the induction of E-cadherin expression. Determining whether there is DNA methylation transferase in breast cancer also plays an important role in regulating ADCY6 DNA methylation is an important direction for future research (44).

In recent years, increasing evidence has shown that the posttranscriptional regulation of genes plays a key role in the occurrence and development of tumors, and the most important posttranscriptional regulation is through the combination of microRNA (miRNA) and targeted mRNA to induce targeted mRNA degradation or translation inhibition (45–47). Studies have shown that microRNA expression disorders and mutations can be found in various diseases, and are believed to be related to the occurrence and development of tumors (48, 49). Another significant finding of our study was that miR-27a-3p directly targeted the 3’ UTR of TET1 and subsequently regulated the expression of ADCY6 and EMT. MiR-27a-3p directly binds to SLC7A11 3’-UTR, resulting in downregulation of SLC7A11 expression and regulation of ferroptosis in NSCLC cells (50). Analysis of clinical data found that miR-27a-3p was downregulated in HCC tissues and correlated with metastasis, Child–Pugh grade and ethnicity. In addition, miR-27a-3p may increase the sensitivity of liver cancer to cisplatin by regulating PI3K/Akt signalling pathway (51). In breast cancer, the expression level of LPAR6 is significantly downregulated, and miR-27a-3p can positively regulate the expression of LPAR6 and affect the function of cell cycle signalling pathway (52). Previous studies have also shown that miR-27a-3p can target and negatively regulate MAGI2, which inactivates the PI3K/AKT signalling pathway by upregulating PTEN and downregulating PD-L1, thereby promoting immune evasion in breast cancer cells (53). Our results were consistent with these reports that miR-27a-3p is upregulation in breast cancer. In addition, we found that the decrease in ADCY6 caused by the degradation of TET1 induced by miR-27a-3p overexpression. More importantly, the increase in ADCY6 reversed the promoting effect of miR-27a-3p overexpression on the malignant biological behaviour of breast cancer cells and reduced the expression of EMT-related proteins that had been increased by miR-27a-3p. These results further support that miR-27a-3p regulates ADCY6 expression by targeting TET1, and can significantly affect EMT.

In conclusion, our study demonstrates that TET1, which is negatively regulated by miR-27a-3p, functions as a key enzyme of DNA demethylation for ADCY6 and thereby leads to EMT and regulates malignant biological behaviour of breast cancer. Our findings will provide new directions and ideas for epigenetic research on breast cancer.

## Data availability statement

The original contributions presented in the study are included in the article/**Supplementary Material**. Further inquiries can be directed to the corresponding author.

## Ethics statement

All clinical studies were allowed by the ethics committees of West China Hospital of Sichuan University, and all patients agreed that their tissues would be used for experimental purposes, and all signed a written informed consent form. The patients/participants provided their written informed consent to participate in this study. All protocols of animal experiments were approved by the Animal Ethics Committee of West China Hospital of Sichuan University.

## Author contributions

HW and QL: manuscript editing, review, and data analysis. JQ, ZW, TH, and CZ: data collection and experiments. All authors read and approved the final manuscript.

## Funding

This study was supported by the National Natural Science Foundation of China (No. 82100655), the Technology innovation research and development project of Chengdu Science and Technology Bureau (2019-YF05-01082-SN), Key research and development projects of Sichuan Science and Technology Department (22QYCX0129), Key research and development projects of Sichuan Science and Technology Department (22ZDYF1209).

## Conflict of interest

The authors declare that the research was conducted in the absence of any commercial or financial relationships that could be construed as a potential conflict of interest.

## Publisher’s note

All claims expressed in this article are solely those of the authors and do not necessarily represent those of their affiliated organizations, or those of the publisher, the editors and the reviewers. Any product that may be evaluated in this article, or claim that may be made by its manufacturer, is not guaranteed or endorsed by the publisher.

## Supplementary material

Supplementary Figure 1 The expression of ADCY6 in TCGA and CPTAC databases. **(A)** The mRNA expression of ADCY6 in TCGA database. (**B)** The protein expression of ADCY6 in CPTAC database. (**C)** The mRNA expression of ADCY6 was analysed by race in TCGA database.

Supplementary Figure 2 MiR-27a-3p expression is increased in breast cancer samples and cell lines. **(A)** The expression of miR-27a-3p in breast cancer samples. N = 56. *p < 0.05 versus the adjacent normal tissues. (**B)** The expression of miR-27a-3p in breast cancer cell lines. *p < 0.05 versus MCF-10 cells.

Supplementary Figure 3 MiR-27a-3p downregulation markedly suppressed proliferation, migration and invasion in MDA-MB-231 and MCF-7 cells. **(A)** The expression of miR-27a-3p after decreasing the expression of miR-27a-3p in MDA-MB-231 cells. *p < 0.05 versus controls. **(B)** The expression of miR-27a-3p after decreasing the expression of miR-27a-3p in MCF-7 cells. *p < 0.05 versus controls. **(C)** The uptake capacity of EdU in MDA-MB-23 cells that decreased the expression of miR-27a-3p was determined by EdU assay. Scale bars, 50 µm. *p < 0.05 versus controls. **(D)** The uptake capacity of EdU in MCF-7 cells that decreased the expression of miR-27a-3p was determined by EdU assay. Scale bars, 50 µm. *p < 0.05 versus controls. **(E)** The proliferation of MDA-MB-231 cells that decreased the expression of miR-27a-3p was determined by CCK-8 assay. *p < 0.05 versus controls. **(F)** The proliferation of MCF-7 cells that decreased the expression of miR-27a-3p was determined by CCK-8 assay. *p < 0.05 versus controls. **(G)** The migration of MDA-MB-231 cells that decreased the expression of miR-27a-3p was determined by Transwell assay. *p < 0.05 versus controls. **(H)** The migration of MCF-7 cells that decreased the expression of miR-27a-3p was determined by Transwell assay. *p < 0.05 versus controls. **(I)** The invasion of MDA-MB-231 cells that decreased the expression of miR-27a-3p was determined by Transwell assay. *p < 0.05 versus controls. J The invasion of MCF-7 cells that decreased the expression of miR-27a-3p was determined by Transwell assay. *p < 0.05 versus controls.

Supplementary Figure 4 The expression of ADCY6 and EMT components was detected by immunohistochemistry. **(A)** The protein expression of ADCY6 and EMT components, including E-cadherin, N-cadherin, vimentin, twist1 and Ki-67 in MDA-MB-231-De-miR-27a-3p cells after decreased the expression of ADCY6 was detected by immunohistochemistry. Scale bars, 25 µm. **(B)** The protein expression of ADCY6 and EMT components, including E-cadherin, N-cadherin, vimentin, twist1 and Ki-67 in MCF-7-De-miR-27a-3p cells after decreased the expression of ADCY6 was detected by immunohistochemistry. Scale bars, 25 µm.

Supplementary Figure 5 Effect of reducing the expression of miR-27a-3p on EMT. **(A)** Protein expression of EMT components, including: E-cadherin, N-cadherin, vimentin, twist1 and Ki-67 in MDA-MB-231cells. *p < 0.05 versus controls. **(D)** Protein expression of EMT components, including: E-cadherin, N-cadherin, vimentin, twist1 and Ki-67 in MCF-7 cells. *p < 0.05 versus controls.

Supplementary Figure 6 Effect of reducing the expression of TET1 on EMT in MDA-MB-231-De-miR-27a-3p and MCF-7-De-miR-27a-3p cells. **(A)** Protein expression of TET1, ADCY6 and EMT components, including: E-cadherin, N-cadherin, vimentin, twist1 and Ki-67 in MDA-MB-231-De-miR-27a-3p cells after decreased the expression of TET1. *p < 0.05 versus controls. **(D)** Protein expression of TET1, ADCY6 and EMT components, including: E-cadherin, N-cadherin, vimentin, twist1 and Ki-67 in MCF-7-De-miR-27a-3p cells after decreased the expression of TET1. *p < 0.05 versus controls.
