# Supplementary figures and images for: MiR-27a-3p binds to TET1 mediated DNA demethylation of ADCY6 regulates breast cancer progression via epithelial-mesenchymal transition
Source: Front Oncol. 2022 Aug 1;12:957511. doi: 10.3389/fonc.2022.957511 (PMC9377375; doi:10.3389/fonc.2022.957511)

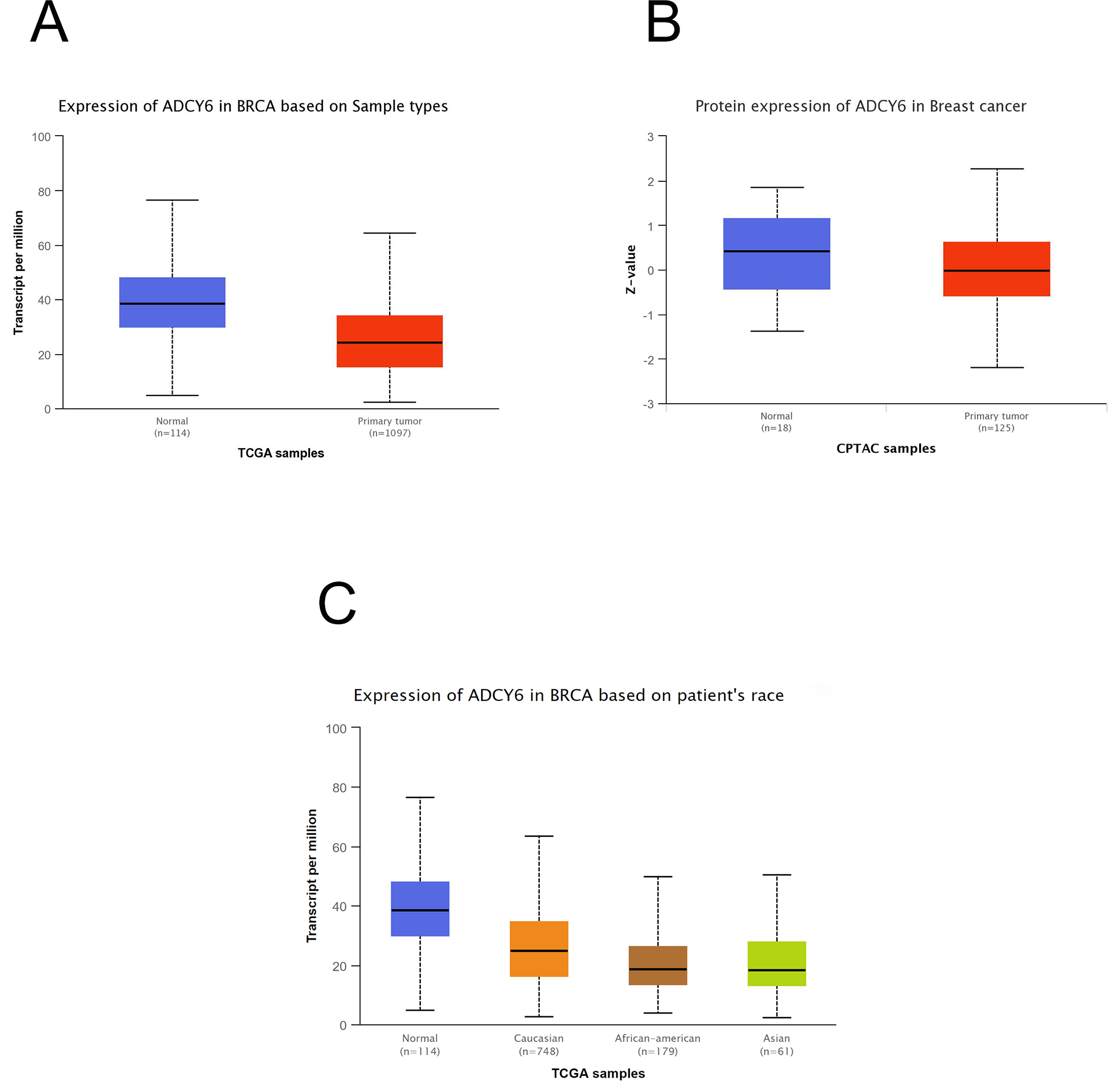

Supplement: Supplementary Figure 1 — The expression of ADCY6 in TCGA and CPTAC databases. (A) The mRNA expression of ADCY6 in TCGA database. (B) The protein expression of ADCY6 in CPTAC database. (C) The mRNA expression of ADCY6 was analysed by race in TCGA database. [file DataSheet_1.zip › Supplementary Figure 1.JPEG]

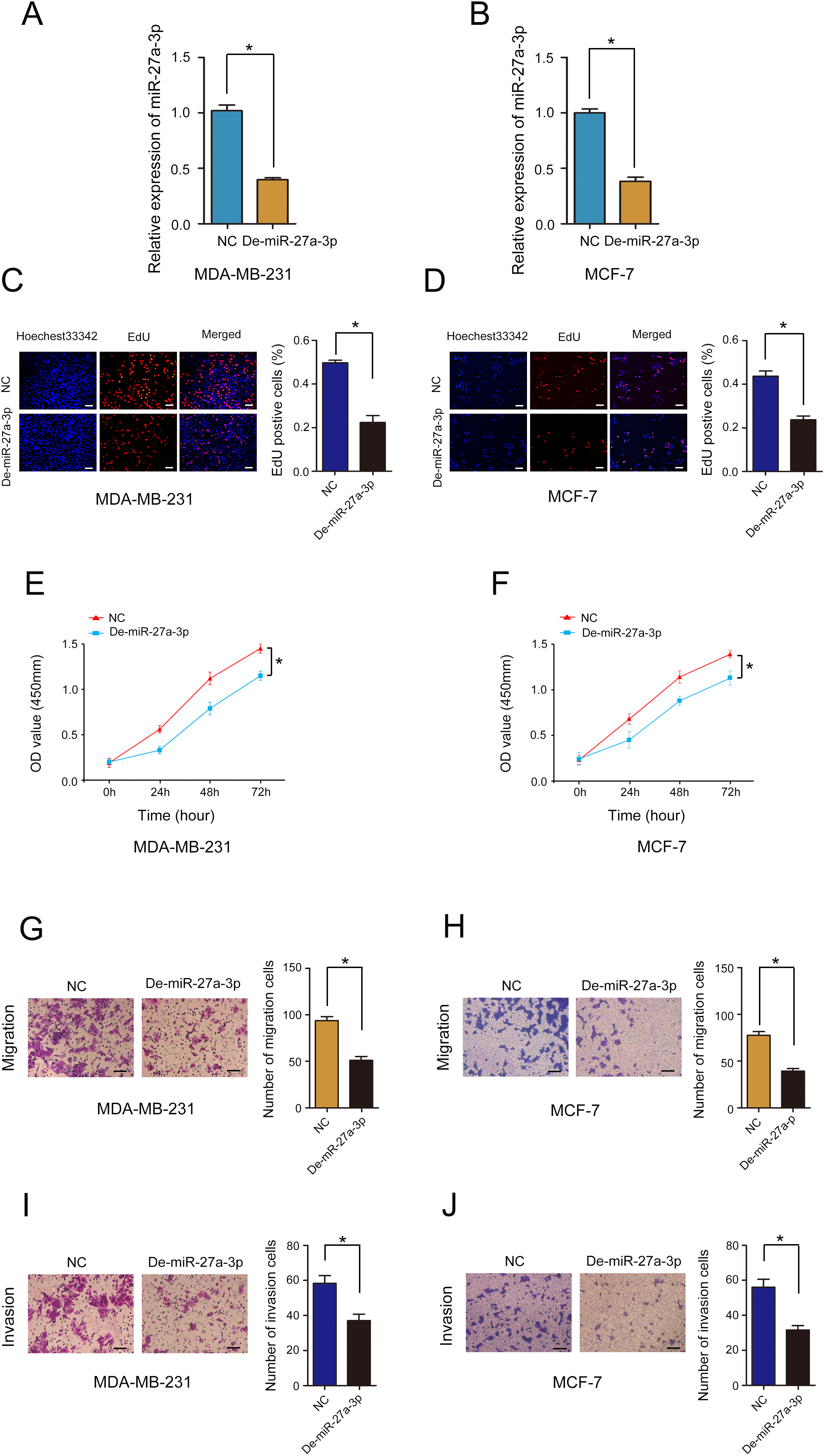

Supplement: Supplementary Figure 1 — The expression of ADCY6 in TCGA and CPTAC databases. (A) The mRNA expression of ADCY6 in TCGA database. (B) The protein expression of ADCY6 in CPTAC database. (C) The mRNA expression of ADCY6 was analysed by race in TCGA database. [file DataSheet_1.zip › Supplementary Figure 2.JPEG]

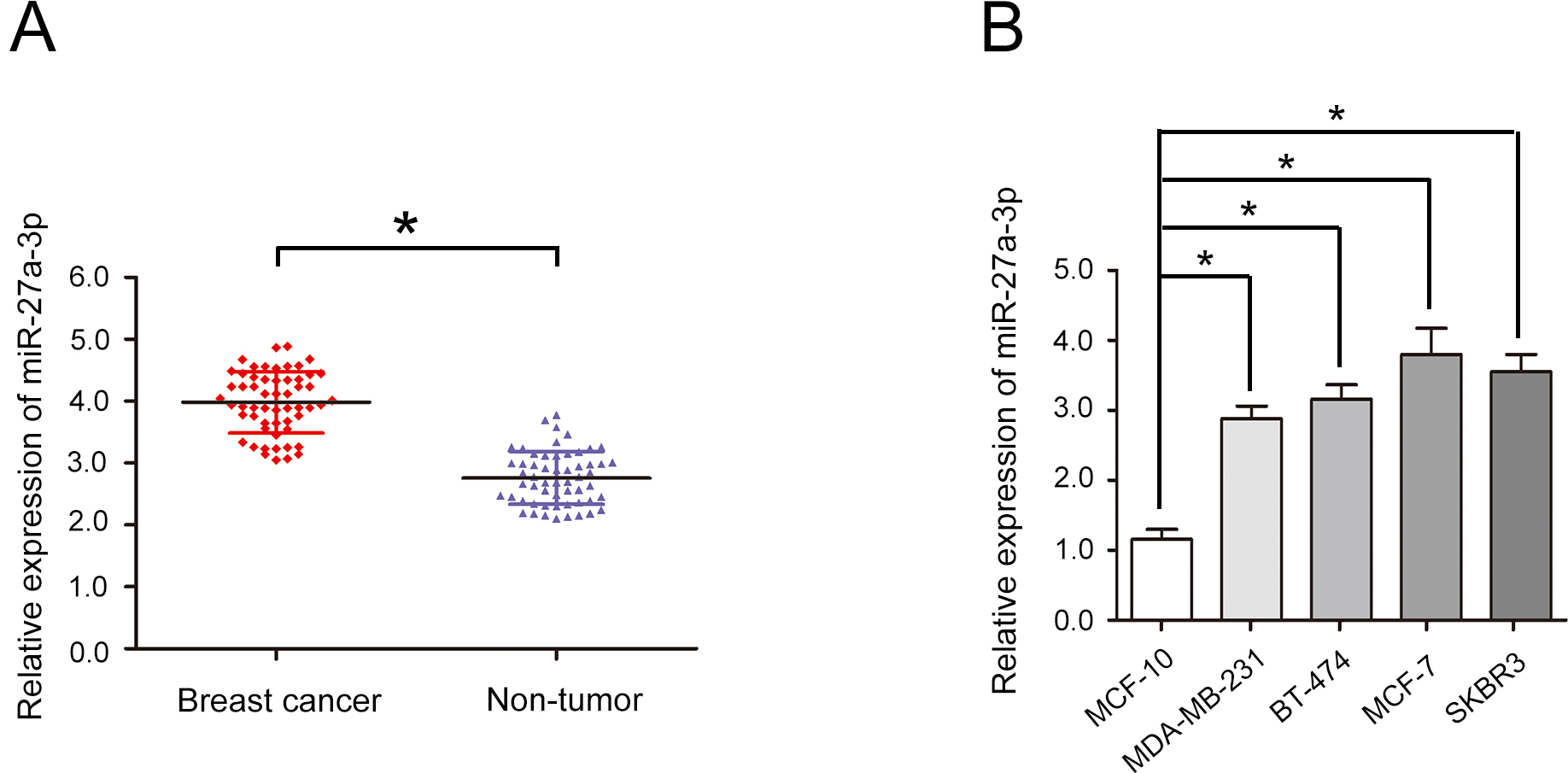

Supplement: Supplementary Figure 1 — The expression of ADCY6 in TCGA and CPTAC databases. (A) The mRNA expression of ADCY6 in TCGA database. (B) The protein expression of ADCY6 in CPTAC database. (C) The mRNA expression of ADCY6 was analysed by race in TCGA database. [file DataSheet_1.zip › Supplementary Figure 3.JPEG]

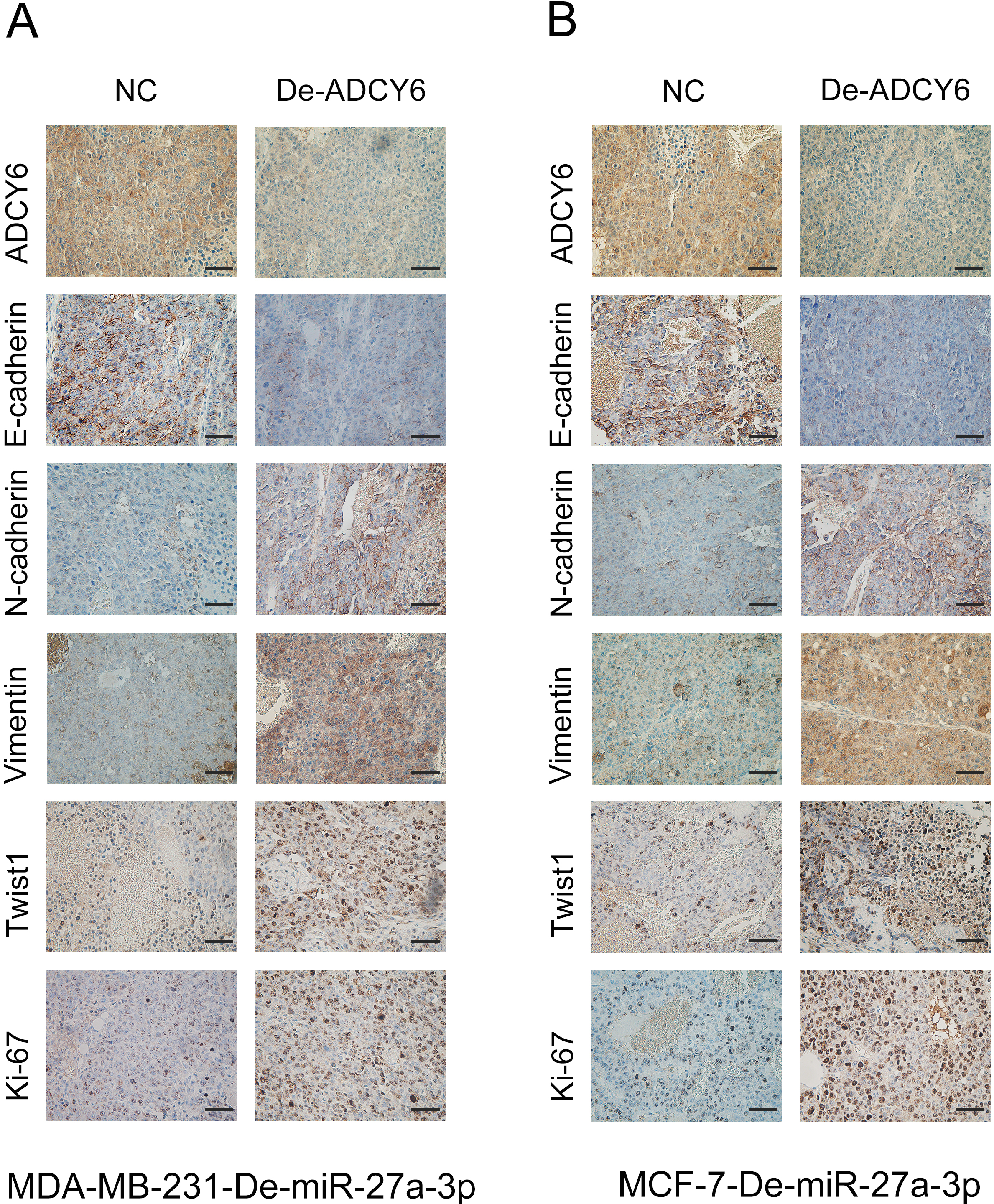

Supplement: Supplementary Figure 1 — The expression of ADCY6 in TCGA and CPTAC databases. (A) The mRNA expression of ADCY6 in TCGA database. (B) The protein expression of ADCY6 in CPTAC database. (C) The mRNA expression of ADCY6 was analysed by race in TCGA database. [file DataSheet_1.zip › Supplementary Figure 4.JPEG]

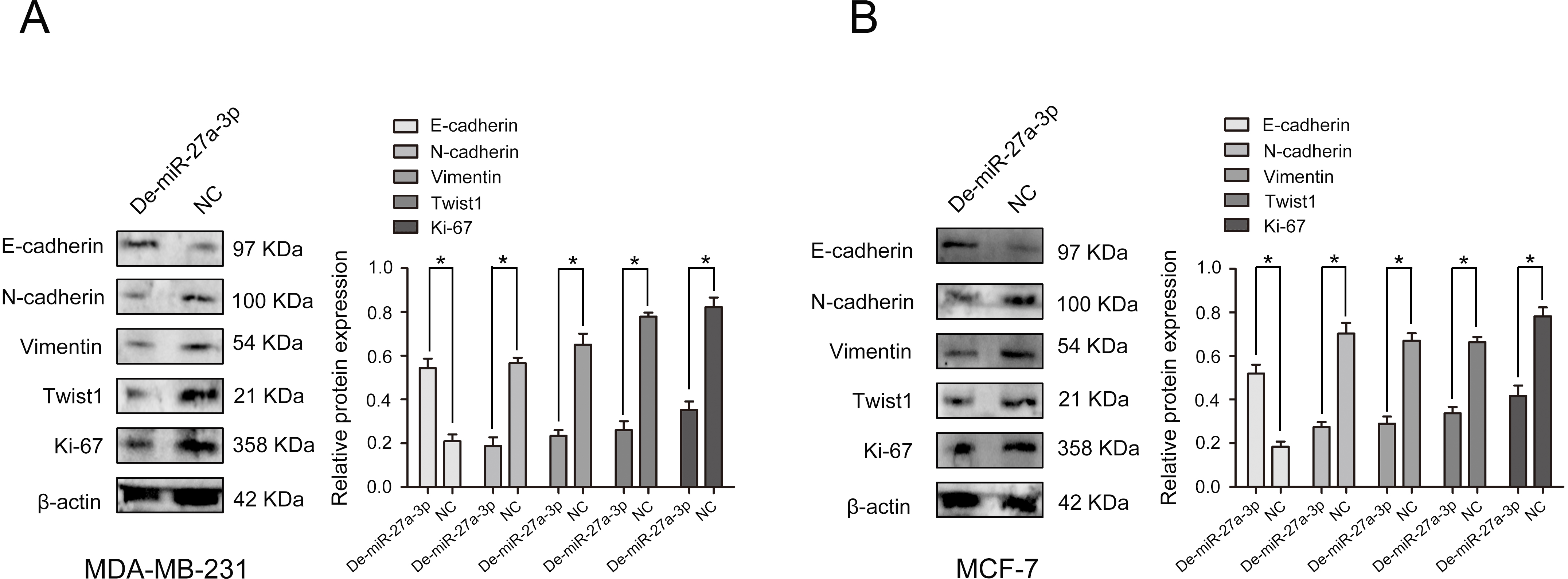

Supplement: Supplementary Figure 1 — The expression of ADCY6 in TCGA and CPTAC databases. (A) The mRNA expression of ADCY6 in TCGA database. (B) The protein expression of ADCY6 in CPTAC database. (C) The mRNA expression of ADCY6 was analysed by race in TCGA database. [file DataSheet_1.zip › Supplementary Figure 5.JPEG]

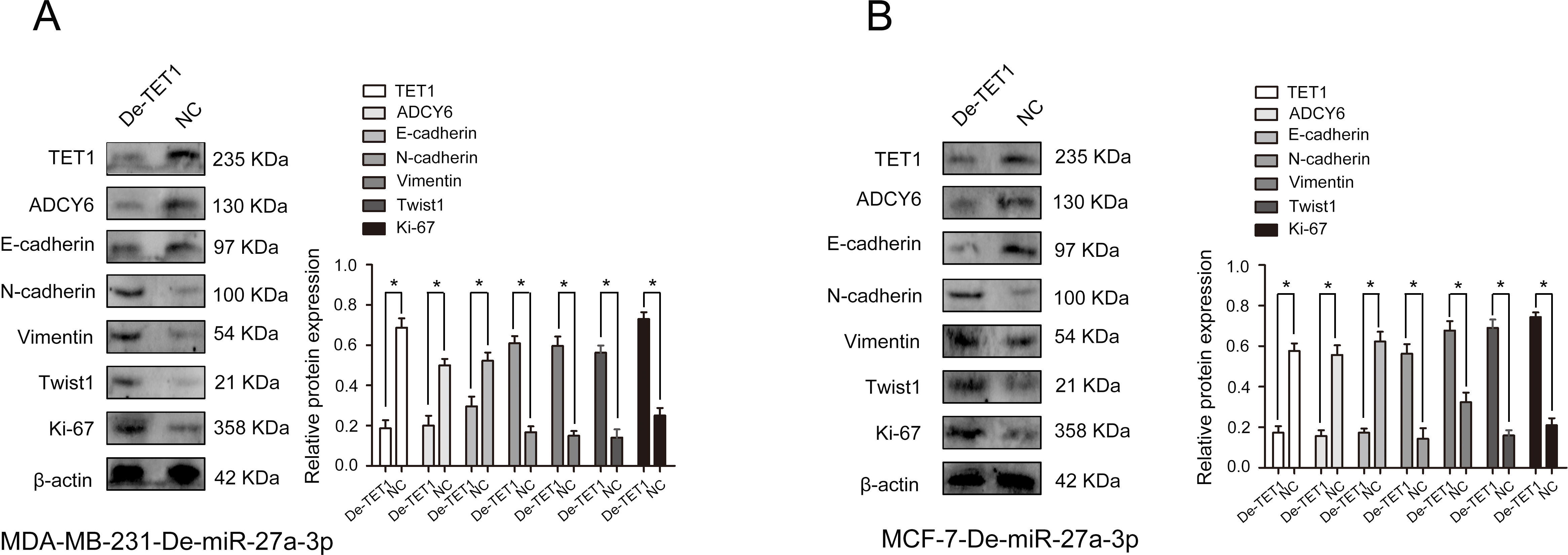

Supplement: Supplementary Figure 1 — The expression of ADCY6 in TCGA and CPTAC databases. (A) The mRNA expression of ADCY6 in TCGA database. (B) The protein expression of ADCY6 in CPTAC database. (C) The mRNA expression of ADCY6 was analysed by race in TCGA database. [file DataSheet_1.zip › Supplementary Figure 6.JPEG]
