# Supplementary material for: MiR-27a-3p binds to TET1 mediated DNA demethylation of ADCY6 regulates breast cancer progression via epithelial-mesenchymal transition
Source: Front Oncol. 2022 Aug 1;12:957511. doi: 10.3389/fonc.2022.957511 (PMC9377375; doi:10.3389/fonc.2022.957511)
Supplement: Supplementary file 2 [file Table_1.docx]

| Cell line | Primary tumor | Estrogen receptors | Progesterone receptors | Human Epidermal GrowthFactor Receptor 2 |
| --- | --- | --- | --- | --- |
| SKBR3 | Invasive ductal carcinoma | No | No | Yes |
| MDA-MB-231 | Invasive ductal carcinoma | No | No | No |
| MCF-7 | Invasive ductal carcinoma | Yes | Yes | No |
| BT-474 | Invasive ductal carcinoma | No | Yes | Yes |
| MCF-10 | Mammary epithelial cells | No | No | No |

**Supplementary table 1** Molecular typing of breast cancer cell lines
